# Alabama State Medical Association

**Published:** 1874-05

**Authors:** 


### ALABAMA STATE MEDICAL ASSOCIATION.

The Sixth Annual Session of this body convened in Selma, on Monday, April 13th, Dr. George A. Ketchum, of Mobile, President, in the chair.

We regret that our efforts to secure a fair *resume* of the proceedings have been unsuccessful. We will endeavor however to give what we can collect from the meagre newspaper reports of the meetings.

"The State Medical Association assembled in this city on Monday last, with a good attendance, and was industriously engaged in the consideration of the weighty matters that demanded its attention until its adjournment, Wednesday night. We have rarely seen a more intellectual looking body of men. The profession in the State is made up of exceptionally good men, and the Association is composed of the pick of the profession. Many of the physicians now here would rank very high anywhere, in this country or in other countries.

"There were present on assembling, Dr. Geo. A. Ketchum, President, Dr. E. A. Semple, Vice-President, and Drs. Jerome Cochran, R. F. Michel, (ex-President,) and Geo. E. Kumpe, censors, and a constitutional quorum of members.

"The Association was welcomed to Selma by Dr. Kent, in behalf of the Selma Medical Association, in a short, neat, wel-conceived, happily expressed and handsomely delivered address.

"Following this welcome came the Annual Address of the President, a carefully prepared and brilliantly written paper of great length, after which the Association entered in a business-like way upon the work of the session.

"On Tuesday night, the Methodist church was densely crowded with a most intelligent audience of ladies and gentlemen to hear the Annual Oration by Dr. Seelye, of Montgomery, who treated an important and practical subject in a most interesting and effective manner.

"Very many interesting papers have been read before the Association, and next volume of its transactions will doubtless be of more interest and more value to the profession than any one that has preceded it.

"The most interesting feature of the proceedings yesterday evening were the reports of Dr. R. F. Michel, of Montgomery, on the yellow fever epidemic in that city last summer; of Dr. Jerome Cochron, of Mobile, on the epidemic of yellow fever, and of Dr. W. H. Anderson, of Mobile, on dengue.

"These gentlemen all agreed that yellow fever and dengue were distinct diseases, of an entirely different nature; that dengue was a nervous disease, and yellow fever a blood disease. They (Drs. Michel and Cochran) considered the latter disease to have been carried to Montgomery and Mobile; that its cause was portable.

"Interesting and able papers were read on the Contribution of Physics and Chemistry to Practical Medicine, by Dr. Thos. Somers, Jr., of Greensboro; on Hemorrhage and Malarial Fever in Alabama, by E. D. McDonald, of Carden; on State Patronage of Asylums and Hospitals, by P. Bryce, of Tuscaloosa; on the Recent Epidemic of Cholera in Birmingham, by M. H. Jordan, of Birmingham; on Puerperal Convulsions, by F. M. Peterson, of Greensboro; Report of a Gynæcological Case, by J. S. Weatherly, of Montgomery. The day's session was consumed by these reports.

"The Association adjourned at midnight Wednesday, after having fixed upon Montgomery as the place for its next meeting, and electing the following officers for the ensuing year: J. S. Weatherly, of Montgomery, President; J. J. Dement, of Huntsville, First Vice-President; R. D. Webb, of Sumter, Second Vice-President; B. H. Riggs, of Selma, Secretary; W. C. Jackson, of Montgomery, Treasurer; J. B. Cochran, of Mobile, James Guild, of Tuscaloosa, R. F. Michel, of Montgomery, G. E. Kumpe, of Leighton, and N. D. Richardson, of Athens, Censors; T. O. Summers, of Greensboro, Orator; and W. A. Johnson, of Uniontown, Alternate Orator."

